# The complete chloroplast genome sequence of the medicinal plant *Fagopyrum dibotrys* (Polygonaceae)

**DOI:** 10.1080/23802359.2018.1483761

**Published:** 2018-09-10

**Authors:** Yan Zhang, Chen Chen

**Affiliations:** Institute of Botany of Shaanxi Province, Xi’an Botanical Garden of Shaanxi Province, Xi’an, Shaanxi Province, China

**Keywords:** Fagopyrum dibotrys, Illumina sequencing, chloroplast genome

## Abstract

Chloroplast (cp) genome sequences become a useful popular tool for population and phylogeny in recent reports. Here, the complete chloroplast genome of the *Fagopyrum dibotrys* has been reconstructed from the whole-genome Illumina sequencing data. The circular genome is 159,325 bp in size, and comprises a pair of inverted repeat (IR) regions of 67,788 bp each, a large single-copy (LSC) region of 84,593 bp, and a small single-copy (SSC) region of 6,944 bp. The total Guanine and Cytosine (GC) content is 38.0%, while the corresponding values of the LSC, SSC, and IR region are 36.3%, 34.5%, and 40.2%, respectively. The chloroplast genome contains 131 genes, including 94 protein-coding genes, eight ribosomal RNA genes, and 29 transfer RNA genes. The Maximum-Likelihood Phylogenetic analysis showed a strong sister relationship with *F. tataricum* in Polygonaceae. Our findings provide a foundation for further investigation of cp genome evolution in *F. dibotrys* and other higher plants.

The chloroplast is necessary organelle in plant with autonomously replicating DNA genome and functions in photosynthesis and bio-synthesis of starch, fatty acids, and other crucial proteins (Hiratsuka et al. 1989). In general, chloroplast genome has typical quadripartite structure consisting of two repeat regions (IRa and IRb), LSC and SSC (Williams et al. 2016).

The perennial herb *Fagopyrum dibotrys* (Polygonaceae), native to southwestern China, is an important medicinal plant. The extraction from the root of *F. dibotrys* has significant pharmacological activities, such as anti-tumour, anti-inflammatiory, and antibacterial (Panda et al. 2011; Liu et al. 2013; Jing et al. 2016). Here, to facilitate its genetic studies, we assembled its chloroplast genome using high-throughput Illumina sequencing technology, as well as analysed its phylogenetic evolution, which will be helpful for further studies on its molecular breeding and genetic engineering.

The DNA samples were extracted from the fresh leaves that were collected from a single individual of *F. dibotrys* in Xi’an Botanical Garden (N34°12’36”, E108°57’15”) and stored in our lab. High-throughput DNA sequencing was conducted on the Illumina HiSeq 2500 Sequencing System (Illumina, San Diego, CA) by Breeding Biotechnologies (Breeding, Yangling, China). Total 24.10 M raw reads were retrieved and trimmed by CLC Genomics Workbench v8.0 (CLC Bio, Aarhus, Denmark). A subset of 11.32 M trimmed reads were used for reconstructing the chloroplast genome by NOVOPlasty (Dierckxsens et al. 2016), with that of its congener *Fagopyrum esculentum* (GenBank: NC_010776.1) as the initial reference genome. A total of 723,322,100 individual chloroplast reads yielded an average coverage of 523.5-fold. The chloroplast genome was annotated in GENEIOUS R9 (Biomatters Ltd., Auckland, New Zealand) by aligning with that of *F. esculentum* (NC_010776.1) and was drawn to the circular chloroplast genome sequence map of OGDRAW 1.1.

The chloroplast genome of *F. dibotrys* is a circular DNA molecule with 159,325 bp in size (MH196562). It comprises a pair of inverted repeat (IR) regions of 67,788 bp each, separated by a large single-copy (LSC) region of 84,593 bp and a small single-copy (SSC) region of 6,944 bp. The total GC content is 38.0%, while the corresponding values of the LSC, SSC, and IR region are 36.3%, 34.5%, and 40.2%, respectively.

This chloroplast genome harbours 131 functional genes, including 94 protein-coding genes (PCGs), 29 tRNA genes, and eight rRNA genes. Among them, 44 are involved in photosynthesis and 58 genes are involved in self replication. Of PCGs, 64 are located in LSC, 12 in the SSC, and 9 were duplicated in the IR region. All the rRNA genes were located in IR regions. Moreover, among all the protein-coding genes, 14 genes contain one intron, while *ycf3* harbors two introns. This is similar to those previously reported for the chloroplast genomes of most other vascular plants (Chumley et al. 2006).

A total of 48 PCGs sequences among 33 chloroplast genomes were aligned by MAFFT (Katoh et al. 2002) and then were connected as gene strings. The Maximum-Likelihood phylogenetic tree of *F. dibotrys* was generated using those gene strings sequence by MEGA 6.0 (Tamura et al. 2013) with using 500 bootstrap replicates (Figure 1). The phylogenetic analysis showed the position of *F. dibotrys* was situated as the sister of *F. tataricum* in Polygonaceae. Our findings provide a foundation for further investigation of chloroplast genome evolution in *F. dibotrys* and other higher plants.

**Figure 1.**
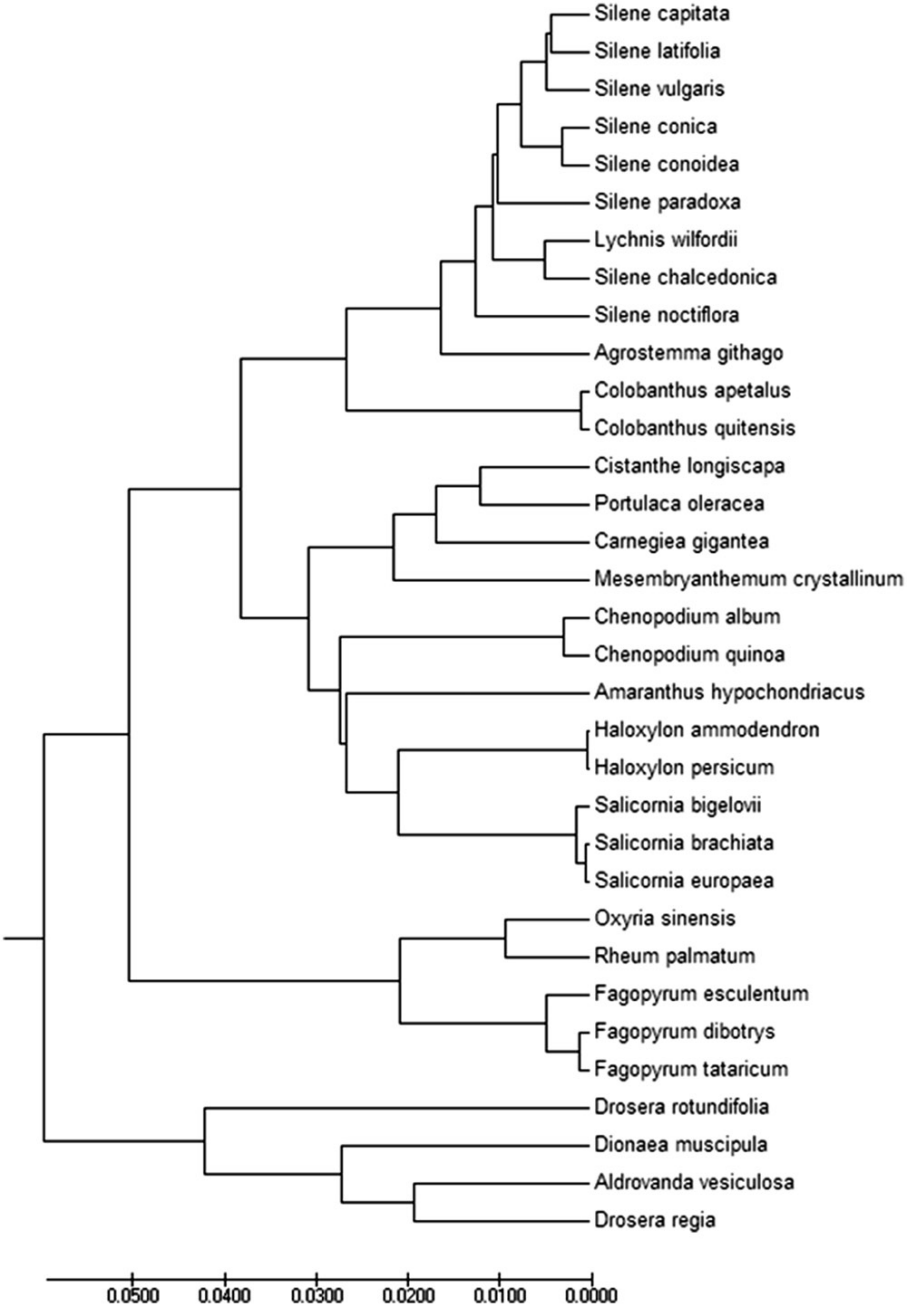
Phylogenetic of 33 species within the family Celastraceae based on the Maximum-Likelihood analysis of the whole chloroplast genome sequences using 500 bootstrap replicates. The analysed species and corresponding Genbank accession numbers are as follows: *Agrostemma githago* (NC_023357.1), *Amaranthus hypochondriacus* • (NC_030770.1), *Aldrovanda vesiculosa* (NC_035416.1), *Carnegiea gigantea* (NC_027618.1), *Chenopodium album* (NC_034950.1), *Chenopodium quinoa* (NC_034949.1), *Cistanthe longiscapa* (NC_035140.1), *Colobanthus apetalus* (NC_036424.1), *Colobanthus quitensis* (NC_028080.1), *Dionaea muscipula* (NC_035417.1), *Drosera regia* (NC_035415.1), *Drosera rotundifolia* (NC_029770.1), *Fagopyrum esculentum* (NC_010776.1), *Fagopyrum tataricum* (NC_027161.1), *Haloxylon ammodendron* (NC_027668.1), *Haloxylon persicum* (NC_027669.1), *Lychnis wilfordii* (NC_035225.1), *Mesembryanthemum crystallinum* (NC_029049.1), *Oxyria sinensis* (NC_032031.1), *Portulaca oleracea* (NC_036236.1), *Rheum palmatum* (NC_027728.1), *Salicornia bigelovii* (NC_027226.1), *Salicornia brachiata* (NC_027224.1), *Salicornia europaea* (NC_027225.1), *Silene capitata* (NC_035226.1), *Silene chalcedonica* (NC_023359.1), *Silene conica* (NC_016729.1), *Silene conoidea* (NC_023358.1), *Silene latifolia* (NC_016730.1), *Silene noctiflora* (NC_016728.1), *Silene paradoxa* (NC_023360.1), *Silene vulgaris* (NC_016727.1).
